# Evaluating the Rumen Degradation of Novel Protected Gelatin Capsules Containing Fish Oil Fed to Lactating Dairy Cows

**DOI:** 10.3390/ani13162555

**Published:** 2023-08-08

**Authors:** Omar Manuel Pena, Kevin Murphy, Nathan Long, Gustavo J. Lascano, Thomas C. Jenkins, Matías J. Aguerre

**Affiliations:** 1 Department of Animal and Veterinary Sciences, Clemson University, Clemson, SC 29634, USA; openape@g.clemson.edu (O.M.P.); nlong2@clemson.edu (N.L.); glascan@clemson.edu (G.J.L.); tjnkns@clemson.edu (T.C.J.); 2 Virtus Nutrition LLC, Corcoran, CA 93212, USA; kmurphy@omegabalancer.com

**Keywords:** rumen protection, EPA, DHA, milk fat

## Abstract

**Simple Summary:**

An increased intake of polyunsaturated fatty acids has been associated with beneficial effects in human health such as a reduction in susceptibility to cardiovascular and autoimmune diseases. However, due to rumen microbial processes, most of the polyunsaturated fatty acids fed to ruminants are changed to saturated forms that the cow can use. Thus, only a small percentage of the fatty acids contained in the cow’s diet reach the intestine and are further incorporated into milk and meat. In this study we conducted two trials to evaluate the effect of feeding rumen-protected capsules containing fish oil on lactation performance, rumen fatty acids content and milk enrichment of fatty acids in the two major dairy breeds. The results of this study showed that the capsules did not increase the concentration of the desired fatty acids, but they reduced their degradation rate in the rumen. A further test showed that the capsules experienced a reduction in shell hardness when exposed to high levels of moisture, leading to abrasion that decreased the effectiveness of the treatments in the two trials. Thus, future studies are warranted to evaluate alternative methods, such as coating, to minimize the contact between the capsule shell and environmental moisture.

**Abstract:**

The objective of this study was to assess the effects of feeding gelatin capsules containing fish oil, treated with alcoholic solutions of flavoring agents followed by drying, on lactation performance, rumen fatty acids content and milk enrichment of fatty acids. In Trial 1, four multiparous ruminally fistulated Holstein cows were randomly assigned to one of four dietary treatments sequences in a 4 × 4 Latin square design. Treatments consisted of (1) Control with no capsules, (2) Control plus 200 untreated capsules per cow/day, mixed with the TMR, (3) Control plus 200 treated capsules per cow/day placed directly into the rumen, (4) Control plus 200 treated capsules per cow/day, mixed with the TMR. In Trial 2, three fistulated Holstein and three fistulated Jersey multiparous cows were randomly assigned to three dietary treatments sequences in a replicated 3 × 3 Latin square design. Treatments consisted of (1) Control with no capsules fed to the cows, (2) Control plus 180 untreated capsules per cow/day, (3) Control plus 180 treated capsules per cow/day. Compared to control, feeding fish oil capsules significantly (Trial 1) or numerically (Trial 2) reduced milk fat concentration and yield. Furthermore, in both trials, the feeding of untreated or treated capsules had no effect on animal performance or milk composition. In both trials, compared to controls, supplementing the diet with fish oil capsules consistently increased total trans C18:1 isomers and DHA concentration in the rumen and milk fat. However, for both trials, capsule protection treatment had a minimal effect on the concentration of any of the reported rumen and milk fatty acids. When assessed under laboratory control conditions, due to water absorption, the treated capsule weight was increased by 40% while resistance to pressure decreased by 84% after 2 h of incubation in water. The results of this study suggest that due to a reduction in the capsule shell’s resistance to abrasion, treated capsules marginally prevented the release of fish oil in the rumen.

## 1. Introduction

An increased intake of polyunsaturated fatty acids (PUFA) has been associated with multiple beneficial effects in human health, including a reduction in susceptibility to cardiovascular [1] and autoimmune diseases [2]. However, due to the rumen biohydrogenation process, most of the PUFA fed to ruminants is hydrogenated to more saturated forms [3]. Thus, only a small percentage of the PUFA consumed by cattle is absorbed in the intestine and further incorporated into milk and meat. The supplementation of dairy cow diets with fish oil, which contains a large quantity of unsaturated, bioactive n-3 fatty acids (FA) including docosahexaenoic acid (DHA) and eicosapentaenoic acid (EPA), has increased the concentration of *cis*-9, trans-11 CLA and long chain n-3 PUFA in milk [4]. However, DHA supplementation can also increase the levels of trans-18:1 in the rumen with a concomitant decrease in milk fat yield caused by fish oil FA increasing rumen biohydrogenation intermediates that inhibit milk fat synthesis [4,5,6]. Several methods of protecting PUFA from rumen metabolism have been investigated extensively with the objective of improving milk production, reproduction, digestion, metabolism and immunity [7]. For example, calcium salts are often reported to be one of the main commercially practical rumen protection formulations that allow the protection of polyunsaturated FA against rumen biohydrogenation even though their efficacy is debated. Formaldehyde is one of the oldest cross-linking agents used to covalently bond proteins [8]. Heating feeds that contain reactive amines and free sugars have been widely used to reduce the solubility of CP and increase the RUP fraction of various feedstuffs [9]. A similar approach has been evaluated to reduce the rumen fermentation of glucose [10]. However, these methods also have several disadvantages including protection by dissociation in the rumen at pH below 6.3 (calcium salts), toxicity and cost (formaldehyde and heating). In addition, there is a large variation in transfer efficiencies of dietary PUFA to milk, both between and within protection techniques. We propose a novel alternative rumen protection method where the target nutrient is contained within a protein (gelatin) capsule and treated with flavoring agents and heat, which enables resistance to dissolution in ruminal contents but will also allow the gelatin to break down post-ruminally. One of the advantages of this novel method includes the possibility of loading the capsules with multiple nutrients (e.g., essential amino acids, essential FA and pharmaceuticals). In addition, there is no chemical modification of the added nutrients. Recently, Jenkins [11] reported that treating capsules containing fish oil with the aforementioned method reduced their disintegration rate in the ruminal fluid and minimized changes in the fatty acid profile of the fish oil content under in vitro conditions.

Additionally, extensive capsule disintegration occurred that allowed the rapid release of FA when treated capsules were exposed to pancreatic proteases. However, in situ methods cannot replicate the different conditions to which the capsules will be exposed from the time they are mixed in the diet and during their passage through the ruminant gastrointestinal tract. We hypothesized that feeding fish oil in gelatin capsules treated for rumen protection will yield higher concentrations of EPA and DHA in milk than feeding fish oil in untreated gelatin capsules, without impairing animal performance, when fed to lactating dairy cows. For this proof-of-concept study, two trials were conducted in different locations with the objective of assessing the effects of feeding cows untreated and treated gelatin capsules containing fish oil on lactation performance, rumen FA content and milk enrichment of FA.

## 2. Materials and Methods

### 2.1. Animal and Treatments

Trial 1. This study was conducted at the phdR&D Research Institute, located in Fort Atkinson, WI, USA. Four multiparous ruminally fistulated Holstein cows (means ± SD; 140.2 ± 56.6 DIM) were randomly assigned to one of four dietary treatments sequences in a 4 × 4 Latin square design with 21 d periods. Treatments consisted of (1) Control with no capsules (CO), (2) Control plus 200 untreated capsules per cow/day and mixed with the TMR (UF), (3) Control plus 200 per cow/day of treated capsules placed directly into the rumen (TR), (4) Control plus 200 treated capsules per cow/day and mixed with the TMR (TF). Capsules were mixed by hand in the TMR during the morning and afternoon feeding. The number of capsules not consumed by the cows was not recorded in this trial. Each capsule contained 140 mg of EPA, 65 mg of DHA, 16.5 mg of Oleic acid (18:1n9), 10.5 mg of Palmitoleic acid (16:1n7) and 9.5 mg of Docosapentaenoic acid (DPA). Thus, each cow was supplied with 28 g of EPA and 13 g of DHA per day. The rumen-protected capsules used in this study were treated with alcoholic solutions of flavoring agents followed by drying.

The relative proportion of dietary ingredients and the chemical composition of the diet are reported in Table 1. The first 7 days of each period were a washout week with no treatment added to the rations, followed by 7 days of adaptation and sample collection performed during the final week. Cows were housed in tie-stall barns bedded on rubber mats with chopped pine shavings as bedding and had free access to water throughout the experiment. Diets were offered as total mixed rations (TMR) twice daily allowing for 5% refusals. Ingredient mix was adjusted based on forage DM analysis conducted three times per week. The care and handling of animals used for the study was conducted as outlined in the guidelines of the Clemson University Committee on Animal Use (AUP2018-15).

Trial 2. The study was conducted at the LaMaster Dairy Center, Clemson University, Clemson, SC, USA. Three multiparous Holstein (means ± SD; 620 ± 25.0 kg of BW; 155 ± 18 DIM) and three multiparous Jersey (427 ± 7.3 kg of BW; 120 ± 22 DIM) ruminally fistulated lactating cows were randomly assigned to one of three dietary treatments sequences in a replicated 3 × 3 Latin squares design with 21 d periods. Treatments consisted of (1) Control with no capsules fed to the cows (CON); (2) Control plus 180 untreated capsules per cow/day (UC); (3) Control plus 180 treated capsules per cow/day (TC). Capsules were mixed by hand in the TMR during the morning and afternoon feeding. Each capsule contained 91.6 mg of EPA and 75 mg of DHA. The number of capsules not consumed by the cows was recorded daily. On average, cows consumed 170 capsules when fed the UC and TC treatments. Thus, each cow was supplied with 15.58 g of EPA and 12.75 g of DHA per day. 

The relative proportion of dietary ingredients and the chemical composition of the diet are reported in Table 1. Cows were housed in tie-stall barns bedded on rubber mats with chopped wheat straw as bedding and had free access to water throughout the experiment. Diets were offered as total mixed rations (TMR) twice daily allowing for 5 to 10% refusals. Ingredient mix was adjusted based on weekly forage DM analysis. The care and handling of animals used for the study was conducted as outlined in the guidelines of the Clemson University Committee on Animal Use (AUP2019-017).

### 2.2. Sampling and Analyses of Feed and Milk

Trial 1. Samples of the TMR and feed refusals were collected twice per week, immediately dried at 55 °C (forced-air oven) for 48 h, ground to pass a 1 mm Wiley mill screen (Arthur H. Thomas, Philadelphia, PA, USA) and composited by period. Samples were analyzed by Cumberland Valley Analytical Services (Waynesboro, PA, USA) for N (method 990.03; AOAC International, 2006) [12], NDF using α-amylase and sodium sulfite [13], ADF (method 973.18; AOAC International, 2000) [14], ether extract (method 954.02; AOAC International, 2000) [14] and starch [15]. Individual cow DMI was computed weekly based on daily records of TMR offered and refused and the 105 °C DM contents of the TMR and refusals.

Milk production was recorded on each cow at each of the three daily milkings (4 a.m., 12 p.m., 8 p.m.) throughout the study and summarized on a weekly basis. Milk samples were collected on nine consecutive milkings on d 19, 20 and 21 of each period. One sub-sample was collected in a bottle with preservers for analysis and determination of fat and protein by infrared analysis (AgSource Laboratories, Menomonie, WI, USA). A second sub-sample was collected in a bottle with no preserver, composited by cow by day and stored at −20 °C for analysis of FA. The total FA concentration and FA profiles of milk samples were analyzed at Cumberland Valley Analytical Services (Waynesboro, PA, USA). Milk lipids were extracted with hexane:isopropanol, transmethylated in the presence of sodium methoxide and FA and quantified by gas chromatography (Clarus 590 GC, PerkinElmer, Waltham, MA, USA) with a flame ionization detector and a 100 m × 0.25 mm × 0.2 µL Supelco SP-2560 column. The average daily concentration and yield of milk components were computed using three milkings’ production as weighting factor. The yield of fat-corrected milk (FCM) was calculated as 0.4 × milk prod (kg/d) + 15 × (milk prod/100) × milk prod (kg/d) according to NRC (2001) [16].

Trial 2. Daily samples of approximately 0.5 kg of the TMR and feed refusals were collected and stored at −20 °C. Samples of silages and premixes were collected weekly. Feeds, TMR and refusals samples were dried at 55 °C (forced-air oven) for 48 h and ground to pass a 1 mm Wiley mill screen (Arthur H. Thomas, Philadelphia, PA, USA). Samples were analyzed at Cumberland Valley Analytical Services (Waynesboro, PA, USA) for N, NDF, ADF, ether extract and starch concentration as described for Trial 1. Individual cow DMI was computed weekly based on daily records of TMR offered and refused and the 55 °C DM contents of the TMR and refusals.

Milk production was recorded on each cow at each of the two daily milkings (6 a.m., 6 p.m.) throughout the study and summarized on a weekly basis. Milk samples were collected from six consecutives milking on days 18, 19 and 20 of each period. One sub-sample was collected in a bottle with preservers and analyzed for fat and true protein by infrared analysis (Lancaster Dairy Herd Association, Manheim, PA, USA) with a Foss FT6000 (Foss North America Inc., Eden Prairie, MN, USA). A second subsample from each milking was collected in a bottle without preserver and composited by cow and period for FA analyses. Milk samples were analyzed at Cumberland Valley Analytical Services (Waynesboro, PA, USA) for total FA concentration and FA profiles as described for Trial 1. The average daily concentration and yield of milk components were computed using morning and evening milk production as weighting factor. The yield of fat-corrected milk (FCM) was calculated as 0.4 × milk prod (kg/d) + 15 × (milk prod/100) × milk prod (kg/d) according to NRC (2001) [16].

### 2.3. Sampling and Analyses of Rumen Fluid

Trial 1. Rumen fluid samples were collected on day 21 of each period 2 h after feeding and stored at −20 °C until analysis for FA at the Department of Animal Science of Penn State University, PA, USA. Frozen rumen samples were lyophilized (Virtis 3.5 L XL, The Virtis Co., Corcoran, CA, USA) and then ground to approximately <1 mm using a spinning-blade coffee grinder (model 80335R, Hamilton Beach, Glen Allen, VA, USA). Fatty acids were directly methylated with sodium methoxide followed by methanolic HCL and extracted in hexane [17]. The total FA concentration and FA profiles of rumen samples were determined by GC with FID according to Baldin (2018) [18] with 13:0 FFA, 17:1 TG and 19:0 FAME as internal standards. For statistical analyses, FA concentrations from each sampling point were averaged per cow and per period.

Trial 2. Rumen fluid samples were collected from different locations in the rumen at 0 (pre-feeding), 1, 2 and 4 h after feeding on day 21 of each period and stored at −20 °C until analysis for FA. Frozen rumen samples were lyophilized and analyzed for total FA concentration and FA profiles as described for Trial 1. For statistical analyses, FA concentrations from each sampling point were averaged per cow and per period.

### 2.4. Cow Body Weight and Blood Sampling and Analyses

Trial 2. Individual cow body weight (BW) was recorded once at the beginning of the trial and at the end of each period. Blood samples were collected from each cow every 12 h on day 19 of each period via jugular venipuncture using Serum Z/9-mL Luer Monovette collection tubes (Sarstedt Inc., Newton, NC, USA). Blood samples were allowed to clot at room temperature for about 1 h and then stored for 24 h at 4 °C. All blood samples were centrifuged at 2000× *g* at 4 °C for 20 min. Serum was then collected into separate tubes and stored at −20 °C until analysis. Duplicate 1 mL serum aliquots from all steers were lyophilized (HarvestRight, North Salt Lake, UT, USA) and then transmethylated according to Tipton et al. [19]. An internal standard, methyl tricosanoic (C23:0), was incorporated into each sample during methylation. Each sample of fatty acid methyl esters was analyzed using a Shimadzu GC-2014 gas chromatograph equipped with a Shimadzu AOC-20s automatic sampler. Separations were completed using a 60 m high resolution gas chromatography column (Agilent Technologies, Inc., Santa Clara, CA, USA). Samples were run at a split ratio of 10:1. Fatty acids were identified by comparing the retention times of known standards.

### 2.5. Statistical Analysis

Data were analyzed with the mixed procedure of SAS (SAS version 9.4, SAS Institute Inc., Cary, NC, USA). For Trial 1, the statistical model included the effect of the period (1 to 4), the effect of the cow (1 to 4), the treatment effect (1 to 4) and the residual error. All terms were considered fixed, except for cow and the residual error. Treatment effects were evaluated by the following pre-planned orthogonal contrasts: (1) effect of control vs. fish oil capsules (UF + TR + TF), (2) effect of untreated fish oil fed directly into capsule (UF) vs. treated fish oil capsules (TR + TF) and (3) effect of treated fish oil capsule fed directly into rumen (TR) vs. treated fish oil capsule mixed with TMR (TF).

For Trial 2, the model included the effect of square (i.e., breed, 1 to 2), the effect of the period (1 to 3), the cow within square effect (1 to 4), the treatment effect (1 to 3), the interaction between square and treatment and the residual error. All terms were considered fixed, except for cow (within square) and the residual error. Treatment effects were evaluated by the following pre-planned orthogonal contrasts: (1) effect of Control vs. fish oil capsules (UC + TC) and (2) effect of untreated fish oil capsule (UC) vs. treated fish oil capsule (TC). For both trials, significance was declared for *p* ≤ 0.05 and tendency for 0.05 < *p* ≤ 0.10.

## 3. Results and Discussion

One cow was removed from the study during the last experimental week of Trial 1 because of health issues unrelated to the dietary treatments. No samples were taken from her for this period. On Trial 2, one cow, assigned to UC treatment, was removed from the study during the last week of period 1 because of health issues (mastitis) and was replaced with a non-fistulated cow. For the measurements reported below for Trial 2 (Table 3, Table 5 and Table 8), the interaction between capsules treatments and cow breed did not reach significance, and therefore results will be presented as main effects of treatment and main effects of cow breed.

### 3.1. Feed Intake and Performance

In trial 1, compared with CO, we observed a tendency toward a reduction in DMI (29.4 vs. 28.1 kg/d) and FPCM (45.6 vs. 41.5 kg/d) and a significant decrease in fat concentration (3.40 vs. 2.87%) and yield (1.80 1.43 kg/d) when feeding the fish oil capsules (Table 2). When compared with the untreated capsules, feeding either TF or TR tended to increase FCM and fat concentration. Furthermore, fat yield and FCM/DMI were significantly lower for UC compared with both treated capsule feeding methods. Performance variables reported in Table 2 were unaffected by feeding the treated capsules directly into the rumen or by mixing them with the TMR. 

**Table 2:** Effect of dietary treatments on animal performance for Trial 1.

Item	Treatment ^1^				SEM ^2^	*p*-Value ^3^		
	CO	UF	TF	TR		1	2	3
DM intake, kg/d	29.4	28.2	27.6	28.4	1.1	0.07	NS	NS ^6^
Milk, kg/d	49.7	48.3	50.4	50.2	5.5	NS	NS	NS
4% FCM ^4^, kg/d	45.6	38.4	42.7	43.3	3.6	0.09	0.08	NS
Fat, %	3.40	2.60	2.96	3.04	0.13	0.02	0.07	NS
Protein, %	2.96	2.94	2.84	2.82	0.07	NS	NS	NS
Lactose, %	4.59	4.53	4.59	4.58	0.03	NS	0.17	NS
Fat, kg/d	1.8	1.3	1.5	1.5	0.13	0.01	0.02	NS
Protein, kg/d	1.5	1.4	1.4	1.4	0.17	NS	NS	NS
Milk yield/DMI ^5^	1.68	1.68	1.83	1.77	0.08	NS	NS	NS
FCM/DMI ^5^	1.53	1.33	1.55	1.52	0.06	NS	0.05	NS

^1^ CON = Control with no capsule (CO); UF = Control plus 200 untreated capsules per cow/day and mixed with the TMR; TR = Control plus 200 per cow/day of treated capsules placed directly into the rumen; TF = Control plus 200 treated capsules per cow/day and mixed with the TMR. ^2^ Standard error of the mean. ^3^ *p* ≤ 0.2 are shown for the contrasts: 1 = control vs. fish oil capsules (UF + TR + TF); 2 = untreated fish oil fed directly into the rumen (UF) vs. treated fish oil capsules (TR + TF); 3 = treated fish oil capsule fed directly into the rumen (TR) vs. treated fish oil capsule mixed with the TMR (TF). ^4^ 4% fat corrected milk. ^5^ Efficiencies calculated as milk (kg/d) or FCM (kg/d) divided by DMI (kg/d). ⁶ NS = not significant (*p* ≥ 0.20).

In trial 2, DMI intake was not affected by the dietary treatments (Table 3). However, when compared with CON, milk yield was higher when cows were fed fish oil capsules, regardless of capsule treatment. Consequently, a tendency toward an increase in milk/DMI was observed when cows were fed fish oil capsules. Performance variables reported in Table 3 were unaffected by feeding either untreated or treated capsules. 

**Table 3:** Effect of dietary treatments on animal performance for Trial 2.

Item	Treatment ^1^			SEM ^2^	*p*-Value ^3^		Breed ^4^		SEM	*p*-Value ^3^
	CON	UC	TC		1	2	H	J		
DMI, kg/d	21.6	22.1	22.1	0.47	NS ^7^	NS	25.2	18.6	0.57	<0.01
Milk, kg/d	27.8	31.3	30.2	1.9	0.06	NS	35	24.5	2.4	0.04
4% FCM ^5^, kg/d	29.60	31.20	30.45	1.3	NS	NS	33.7	27.1	1.5	0.03
Fat, %	4.77	4.14	4.18	0.39	0.17	NS	3.99	4.73	0.42	NS
Protein, %	3.33	3.24	3.31	0.10	NS	NS	3.16	3.42	0.13	NS
Fat, kg/d	1.26	1.23	1.16	0.08	NS	NS	1.33	1.11	0.07	0.1
Protein, kg/d	0.88	1.00	0.97	0.06	0.13	NS	1.08	0.82	0.07	0.06
Milk yield/DMI ^6^	1.28	1.41	1.36	0.08	0.06	NS	1.38	1.32	0.11	NS
FCM/DMI ^6^	1.35	1.40	1.34	0.09	NS	NS	1.32	1.40	0.07	NS

^1^ CO = Control with no capsules; UC = Control plus 180 untreated capsules per cow/day; TC = Control plus 180 treated capsules per cow/day. ^2^ Standard error of the mean (highest when uneven samples). ^3^ *p* ≤ 0.2 are shown. The contrasts: 1 = Control vs. fish oil capsules (UC + TC); 2 = untreated fish oil capsules (UC) vs. treated fish oil capsule (TC). ^4^ H = Holstein; J = Jersey. ^5^ 4% fat corrected milk. ^6^ Efficiencies calculated as milk (kg/d) or FPCM (kg/d) divided by DMI (kg/d). ^7^ NS = not significant (*p* ≥ 0.20).

The negative effect of feeding fish oil on intake is largely dependent on the amount of oil fed and its characteristics; one of them is the smell produced when it is directly mixed in the diet [20]. However, the tendency toward a reduction in intake observed in Trial 1 was unexpected because the amounts of fish oil fed in the trial were below the ones reported in the literature that might have an impact on feed intake [6]. Furthermore, feeding fish oil capsules will presumably reduce or eliminate the smell as a detrimental factor for DMI. The increase in milk production observed in Trial 2 is also puzzling, as most studies have shown that fish oil supplementation has no effect on milk yield. In Trial 1, adding fish oil capsules to the diet or directly into the rumen decreased milk fat content and yield, which is a typical response that has been frequently reported in the literature [19,20,21], but it was also observed that treated capsules tended to mitigate to some extent the negative effect of feeding fish oil on milk fat concentration and yield. In Trial 2, the lack of a treatment effect on milk percentage and yield suggests that either treated capsules broke in the rumen but the amount of fish oil released was not enough to cause MFD, or the treated capsules did not dissolve in the ruminal fluid but were also not digested in the intestine, as indicated by the profile of fatty acids in blood. We recovered several pieces of treated capsules during ruminal sampling indicating that although the capsule shell did not dissolve, it did not remain intact after ingestion and during rumen passage. Thus, we hypothesize that abrasion in the rumen broke the capsule shells but that the released oil did not penalize milk fat synthesis. Donovan (2000) [20] found no difference in milk fat percentage in cows fed 290 g/d of fish oil compared to cows under no supplementation. Moreover, several authors [20,22,23] observed a negligible fish oil effect when fed at higher doses than the one used in this study. 

As expected, Holstein cows have significantly higher DMI, milk yield and FCM yield than Jersey cows (Table 3). These results were consistent with previous studies comparing both cow breeds when fed a TMR diet [24,25]. The cow breed did not influence feed efficiency, but the concentration of milk fat and protein in Jersey cows was numerically higher than in Holstein cows. The increase in fat and protein content in Jersey cow milk has been frequently reported in the literature [24,25]. Due to the large difference in milk production, the yield of fat and protein tended to be higher for Holstein cows.

### 3.2. Rumen Fluid Fatty Acid Composition

Table 4 presents the effects of the dietary treatments on the total fatty acid concentration in the rumen fluid for Trial 1. Compared with CO, the addition of fish oil capsules to the diet decreased the concentration of C18:0 while numerically increasing *cis*-9 C18:1 by 38 and 10%, respectively. Additionally, feeding untreated or treated fish oil capsules consistently increased trans-6/8 C18:1, trans-9 C18:1, trans-10 C18:1, trans-11 C18:1 and trans-12 C18:1 concentration, compared with CO. Furthermore, most of the increase in total trans C18:1 fatty acid was due to trans-10 C18:1 which along with trans-11 C18:1 is the last intermediate product in two different paths of rumen biohydrogenation of linoleic acid. The increased levels of those fatty acids can be explained by the amount of 18:1n9 present in the capsules. Adding fish oil capsules to the diet has a small impact on *cis*-9 and trans-11 C18:2 CLA concentration. Finally, regardless of the method of administration of the capsules or the protection treatment, feeding fish oil capsules increased DHA content in rumen fluid. However, the amount of DHA detected for untreated capsules was numerically higher compared with treated capsules independent of the delivery method. This finding led us to think that treated capsules were physically stronger and did not release their content in the rumen at the same rate as untreated capsules. The capsule protection treatment or feeding method (rumen vs. feed) had no effect on the concentration of any of the reported rumen FA.

Table 5 presents the effects of the dietary treatments on the total fatty acid concentration in the rumen fluid for Trial 2. As we observed on trial 1, compared with CON, the addition of fish oil capsules to the diet decreased the concentration of C18:0 while tending to increase *cis*-9 C18:1 by 20 and 14%, respectively. Similarly, the concentration of total *trans*-C18:1 FA was increased by 39% when fish oil capsules were added to the diet. Adding fish oil capsules to the diet has no impact on *cis*-9, *trans*-11 C18:2 CLA and *trans*-10, *cis*-12 C18:2 CLA concentration. Compared with CON, DHA and total n-3 concentrations in ruminal fluid were higher when feeding untreated or treated capsules. The capsule protection treatment had no effect on the concentration of any of the reported rumen FA, except for a lower DHA concentration compared with. Rumen fluid collected from Jersey cows had a lower concentration of C14:0, C15:0 and C17:0 and a higher concentration of DHA.

**Table 5:** Rumen FA profile of lactating cows fed the dietary treatments in Trial 2.

FA, g/100 g of Total FA	Treatment ^1^			SEM ^2^	*p*-Value ^3^		Breed ^4^		SEM ^2^	*p*-Value ^3^
	CON	UC	TC		1	2	H	J		
C14:0	0.87	0.84	0.84	0.03	NS ⁷	NS	0.9	0.8	0.02	0.04
C15:0	0.55	0.52	0.50	0.02	0.10	NS	0.55	0.5	0.01	0.07
C16:0	20.9	20.9	20.5	0.35	NS	NS	20.9	20.6	0.31	NS
C17:0	0.40	0.37	0.36	0.01	0.02	NS	0.39	0.36	0.01	0.04
C18:0	35.8	27.9	29.1	1.9	0.03	NS	32.3	29.5	2.00	NS
*trans*-6/8 C18:1	0.47	0.67	0.60	0.04	0.02	NS	0.58	0.59	0.04	NS
*trans*-9 C18:1	0.26	0.37	0.34	0.02	0.01	NS	0.31	0.34	0.03	NS
*trans*-10 C18:1	0.85	1.41	1.00	0.22	NS	NS	1.27	0.9	0.21	NS
*trans*-11 C18:1	2.77	4.30	3.52	0.41	0.04	0.15	3.35	3.71	0.50	NS
*trans*-12 C18:1	0.76	1.04	0.96	0.04	0.01	NS	0.90	0.94	0.04	NS
Total *Trans*	5.12	7.80	6.42	0.62	0.03	0.13	6.4	6.49	0.70	NS
*cis*-9 C18:1	7.5	8.7	8.4	0.41	0.10	NS	7.9	8.6	0.36	NS
*cis*-9, *cis*-12 C18:2n-6	14.2	15.8	15.2	0.77	NS	NS	14.4	15.8	0.67	NS
*cis*-9, *trans*-11 C18:2 CLA	0.09	0.16	0.14	0.04	NS	NS	0.13	0.14	0.04	NS
*trans*-10, cis-12 C18:2 CLA	0.12	0.11	0.08	0.02	0.17	0.15	0.12	0.09	0.02	NS
*cis*-9, *cis*-12, *cis*-15 C18:3n-3	1.86	1.96	1.99	0.01	NS	NS	1.91	1.96	0.08	NS
C20:0	0.59	0.99	1.06	0.04	<0.01	NS	0.83	0.93	0.04	NS
C20:5n-3 EPA	N/D ⁶	0.44	0.23	0.07	NS	NS	0.15	0.29	0.06	NS
C22:6n-3 DHA	0.09	0.46	0.33	0.03	<0.01	0.02	0.20	0.38	0.04	0.04
Total n-3 ^5^	1.94	2.89	2.65	0.19	0.02	NS	2.28	2.71	0.19	0.19

^1^ CO = Control with no capsules; UC = Control plus 180 untreated capsules per cow/day; TC = Control plus 180 treated capsules per cow/day. ^2^ Standard error of the mean (highest when uneven samples). ^3^ *p* ≤ 0.2 are shown. The contrast 1 = Control vs. fish oil capsules (UC + TC); 2 = untreated fish oil capsules (UC) vs. treated fish oil capsule (TC). ^4^ H = Holstein; J = Jersey. ^5^ C18:3n-3 + C20:5n-3 + C22:5n-3 + C22:6n-3. ⁶ N/D = non-detectable values. ⁷ NS = not significant (*p* ≥ 0.20).

Studies supplementing dairy diets with fish oil have also reported a reduction in C18:0 concentration in ruminal digesta in vivo [26] and under in vitro conditions [27] as a result of an incomplete biohydrogenation of unsaturated fatty acid. Furthermore, the increase in ruminal *cis*-9 C18:1 and *trans*-18:1 isomer when fish oil is supplemented to the diet, as we observed in both trials, is well documented in the literature [26,28]. It has been proposed that this increase in *cis*-9 C18:1 and *trans*-C18:1 FA with added DHA and EPA may be caused by inhibiting the reductase activity of ruminal microorganisms [27]. Consequently, the similar changes in fatty acid biohydrogenation that we consistently observed when feeding either the untreated or treated fish oil capsules provides further evidence that the capsule treatments were unsuccessful to prevent the release of fish oil in the rumen. However, the lower concentration of DHA in the rumen fluid of cows fed the TC treatment, compared with UC, suggests that the capsule treatment was partially successful to prevent the release of fish oil in the rumen.

### 3.3. Plasma Fatty Acid Composition

Table 6 presents the effects of dietary treatments on plasma FA for Trial 2. Surprisingly, the fatty acid composition of plasma was not greatly affected by dietary treatments. Compared with CON, feeding fish oil capsules numerically increased the concentration of DHA and reduced *cis*-9 C18:1 and C20:4n-6. Moallem (2013) [29], reported a drastic increase in plasma DHA from cows fed encapsulated fish oil, which indicates successful transfer of this fatty acid from the diet into the blood. However, this higher level of transfer can be explained by the time that the project lasted. In their study cows were fed 4.3 g/d of DHA from day 256 of pregnancy until parturition and 13.5 g/d from there to day 100 postpartum. Longer times of supplementation mean higher amounts of consumption [30].

### 3.4. Milk Fatty Acid Composition

Table 7 presents the effects of the dietary treatments on the total concentration of milk FA for Trial 1. Compared with CO, most of the individual short-chain FA decreased or tended to decrease when adding untreated or treated fish oil to the diet, except for C4:0, which remained unchanged by the treatments. However, compared with CO, milk from cows supplemented with fish oil capsules had a 7.4 and 8.4% lower concentration of C16:0 and C18:0 (tendency), respectively. In addition, dietary treatments changed the concentration of several individual *trans* monoene isomers present in the milk. In particular, the addition of fish oil capsules to the diet increased *trans*-6/8 C18:1, *trans*-9 C18:1, *trans*-10 C18:1, *trans*-11 C18:1 and *trans*-12 C18:1 concentration in the milk. Furthermore, fish oil supplementation increased *cis*-9, *cis*-12 C18:2n-6, *cis*-9, *trans*-11 C18:2 CLA and *cis*-9, *cis*-12, *cis*-15 C18:3n-3 concentration by 10, 69 and 14%, respectively.

Regardless of the delivery method, the content of EPA and DPA in milk fat was 2.1 and 5.5 times higher, respectively, when feeding fish oil capsules. Overall, when compared with the untreated capsules, supplementing treated capsules had no effect on milk FA profile, except for a higher *cis*-9 C18:1 and lower *cis*-9, *trans*-11 C18:2 CLA. Furthermore, milk FA contents were largely unaffected by placing the treated fish oil capsules directly into the rumen or by mixing them with the TMR.

In trial 2, fish oil supplementation had a minor effect on the total concentration of most short and medium chain milk FA (Table 8). However, compared with CON, milk from cows supplemented with fish oil capsules had an 8.8 and 10.2% lower concentration of C15:0 and C16:0, respectively. Like in Trial 1, supplementing cows with fish capsules increased the concentration of *trans*-6/8 C18:1, *trans*-9 C18:1, *trans*-10 C18:1 (numerically), *trans*-11 C18:1 and *trans*-12 C18:1 in the milk. Likewise, adding fish oil capsules to the diet increased *cis*-9 C18:1, *cis*-9, *cis*-12 C18:2n-6, *cis*-9, *trans*-11 C18:2 CLA and *cis*-9, *cis*-12, *cis*-15 C18:3n-3 concentration by 8, 13, 40 and 17%, respectively. Adding untreated or treated fish oil capsules to the diet increased total n-3 FA (C18:3n-3, C20:5n-3, C22:5n-3, and C22:6n-3) content by 38% despite not detecting the same increase in plasma. Most of this increase in n-3 FA was due to the increase in EPA and, to a lesser extent, to DHA. 

**Table 8:** Milk FA profile of lactating cows fed the dietary treatments in Trial 2.

FA, g/100 g of Total FA	Treatment ^1^			SEM ^2^	*p*-Value ^3^		Breed ^4^		*p*-Value ^3^
	CON	UC	TC		1	2	H	J	
C4:0	3.79	4.07	3.84	0.13	0.07	0.04	3.79	4.01	NS
C6:0	1.96	2.07	1.93	0.04	0.12	<0.01	1.86	2.11	0.03
C8:0	1.21	1.28	1.18	0.05	NS ⁶	0.02	1.13	1.30	0.17
C10:0	2.86	2.95	2.73	0.20	NS	0.11	2.7	3.0	NS
C12:0	3.43	3.38	3.21	0.25	NS	NS	3.20	3.48	NS
C14:0	10.7	10.6	10.3	0.24	NS	NS	10.5	10.5	NS
*cis*-9 C14:1	0.86	0.78	0.82	0.07	NS	NS	0.81	0.82	NS
C15:0	0.85	0.76	0.79	0.03	<0.01	0.18	0.78	0.82	NS
C16:0	32.3	28.6	29.4	1.09	<0.01	NS	29.9	30.3	NS
*cis*-9 C16:1	1.44	1.38	1.45	0.05	NS	NS	1.43	1.41	NS
C17:0	0.60	0.56	0.59	0.02	0.11	0.18	0.58	0.59	NS
C18:0	10.6	10.4	10.6	0.43	NS	NS	10.4	10.7	NS
*trans*-6/8 C18:1	0.26	0.40	0.35	0.03	<0.01	NS	0.34	0.33	NS
*trans*-9 C18:1	0.21	0.32	0.29	0.02	<0.01	NS	0.27	0.27	NS
*trans*-10 C18:1	0.42	0.71	0.56	0.12	0.15	NS	0.65	0.47	NS
*trans*-11 C18:1	1.08	1.74	1.54	0.15	<0.01	0.12	1.36	1.56	NS
*trans*-12 C18:1	0.39	0.64	0.59	0.04	<0.01	NS	0.54	0.55	NS
*cis*-9 C18:1	15.9	16.9	17.4	0.54	0.02	NS	17.3	16.2	NS
*cis*-9, *cis*-12 C18:2n-6	2.56	2.94	2.85	0.13	0.06	NS	3.03	2.53	0.05
*cis*-9, *trans*-11 C18:2 CLA	0.46	0.67	0.62	0.06	0.01	NS	0.53	0.58	NS
*cis*-9, *cis*-12, *cis*-15 C18:3n-3	0.29	0.35	0.33	0.02	0.07	NS	0.35	0.29	0.12
C20:0	0.16	0.25	0.28	0.02	<0.01	0.16	0.21	0.25	0.15
*cis*-11 C20:1	0.04	0.11	0.11	0.01	<0.01	NS	0.08	0.09	NS
C20:5n-3 EPA	0.03	0.07	0.06	<0.01	<0.01	0.05	0.05	0.06	0.07
C22:5n-3	0.06	0.08	0.07	0.01	0.03	NS	0.07	0.07	NS
C22:6n-3 DHA	0.05	0.08	0.07	0.02	NS	NS	0.07	0.07	NS
Total n-3 ^5^	0.40	0.58	0.52	0.04	0.01	NS	0.51	0.49	NS

^1^ CO = Control with no capsules; UC = Control plus 180 untreated capsules per cow/day; TC = Control plus 180 treated capsules per cow/day. ^2^ Standard error of the mean (highest when uneven samples). ^3^ *p* ≤ 0.2 are shown. The contrast: 1 = Control vs. fish oil capsules (UC + TC); 2 = untreated fish oil capsules (UC) vs. treated fish oil capsule (TC). ^4^ H = Holstein; J = Jersey. ^5^ C18:3n-3 + C20:5n-3 + C22:5n-3 + C22:6n-3. ⁶ NS = not significant (*p* ≥ 0.20).

The response to fish oil supplementation in milk fat composition is well documented in the literature and is consistent with the changes in milk fatty acid profile observed in Trials 1 and 2 [4,5,28]. For example, the supplementation of EPA and DHA usually results in decreased milk fat levels of C18:0 and *cis*-9 C18:1 due to the incomplete biohydrogenation of polyunsaturated FA in the rumen [4,5]. Furthermore, the higher milk fat concentration of *trans*-C18:1 that we observed in both trials is likely the result of DHA from fish oil enhancing *trans*-C18:1 production from other polyunsaturated FA and not the direct conversion of DHA into *trans*-C18:1 isomers [31]. Finally, several studies have shown that fish oil supplementation consistently elevates milk C20:5n-3, C22:5n-3, and C22:6n-3 concentration. However, the lack of difference in milk EPA and DHA concentration and *trans*-C18:1 between the untreated and treated capsules further demonstrates that the proposed protection treatment failed to prevent the breakdown of the capsules in the rumen.

We observed several significant differences in the composition of milk FA between the two breeds used in this study (Table 8). Data from each breed showed that Jerseys produced significantly higher concentrations of C6:0 than Holsteins. However, Holstein cows produced significantly higher concentrations of *cis*-9, *cis*-12 C18:2n-6 and tended to produce higher concentrations of C20:5n-3 EPA than Jerseys. Several of the fatty acid levels reported for the present study do not agree with previous work comparing Holstein and Jersey cows. For example, there was no difference between the two breeds in the total production of several short- and medium-chain FA (C:4 and C8:0 to C12:0) as reported by White (2001) [24]. Similarly, several studies [25,32] have shown that compared with Jersey, Holstein cows have a higher milk content of mono-unsaturated FA and *cis*-9, *trans*-11 C18:2 CLA, and in Holstein cows that is associated with higher Δ9-desaturase activity in the mammary gland [33]. However, the interpretation of the results from this study should be conducted with caution due to the low number of evaluated cows from each breed, which might have precluded us from detecting significant differences.

### 3.5. Moisture Effect on the Shell Hardness of the Treated Capsules

**Figure 1:**
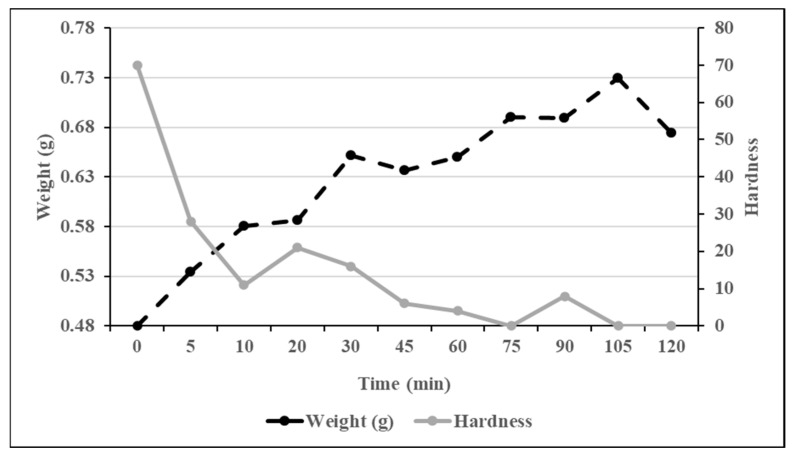
Changes in weight and hardness after incubating treated capsules in water at 39 °C.

During the rumen sampling procedures conducted in Trial 2, the number of rumen intact capsules was visually assessed. We observed several intact and numerous pieces of broken but not degraded treated capsules. However, no intact or pieces of the untreated capsules shells were recovered. Taken together, these observations suggest that the shells of the treated capsules might have suffered a transformation that made them weaker to friction, even though they would not degrade in the rumen. To test this theory, we conducted a trial consisting of submerging 12 treated capsules in a 39 °C water bath for up to 120 min to measure their change in weight and hardness after different incubation lengths (0, 5, 10, 15, 20, 30, 45, 60, 75, 90, 105 and 120 min). One capsule was weighted and evaluated for harness at each time point. Capsule hardness was determined with a durometer (FstDgte, Shore A Durometer, Guilin Digital Electronic Co., Ltd., Guilin, Guangxi, China), which is the international standard to measure the hardness of rubber, plastic and other non-metallic materials. Changes in capsule weight and hardness are illustrated in Figure 1. The smaller capsule weight was measured at time 0 (0.48 g), then steadily increased during the incubation period, and after 120 min the capsule was 40% heavier than at the start of the incubation. On the contrary, the hardness of the capsule was highest at time 0, decline to 84% after only 10 min of incubation, and was negligible after 1 h. These data suggest that when in contact with high levels of moisture, as it is expected when capsules are mixed with the TMR, saliva, and rumen fluid, the shell of the treated capsule was likely weakened, which probably led to the breakup of the capsule due to the abrasion at what it was exposed to in the mouth and the rumen.

## 4. Conclusions

In summary, results from these two studies indicated that gelatin capsules treated with alcoholic solutions of flavoring agents followed by drying containing fish oil for rumen protection did not result in higher concentrations of EPA and DHA in milk compared with untreated gelatin capsules, as we originally hypothesized. In addition, we observed that feeding untreated or treated fish oil capsules consistently increased the production of biohydrogenation intermediates, both in the rumen fluid and in milk fat, with a concomitant detrimental impact on milk fat concentration and yield in one of the trials. In Trial 2, however, the lack of a significant impact on milk fat yield when feeding either the untreated or treated capsules prevents us from making any additional inference on the effectiveness of protection treatment. However, the mitigating effects on milk fat yield observed in Trial 1 when cows were fed treated capsules and the recovered pieces of broken treated shells in Trial 2 confirms the findings of Jenkins et al. [11] which indicated that the proposed protection method reduced the degradation rate of the capsule in the ruminal fluid. Further testing of the capsules revealed the susceptibility of the treated capsules to abrasion because of a reduction in shell hardness when exposed to high levels of moisture. Thus, future studies are warranted to evaluate alternative methods such as coating to minimize the contact between the capsule shell and environmental moisture.

## Figures and Tables

**Table 1:** Ingredient and chemical composition of control diets fed on Trial 1 and Trial 2.

Item	Control Diet	
	Trial 1 (CO)	Trial 2 (CON)
Ingredient composition (%DM)		
Corn Silage	33.3	40.0
Alfalfa Silage	18.6	--
Alfalfa Hay	--	10.0
Soy Hulls	5.3	--
Dry Corn	10.1	--
High Moisture Shell Corn	10.1	--
Whey	3.9	--
Premix Trial 1 ^1^	18.7	--
Premix Trial 2 ^2^	--	50.0
Chemical composition		
DM, as-is	41.2	50.3
CP, %DM	15.5	15.9
NDF, %DM	29.7	33.0
ADF, %DM	20.3	21.2
NFC, %DM	45.0	40.5
Starch, %DM	25.5	27.6
Ether extract, %DM	4.73	3.73
FA, %DM	3.43	--

^1^ Premix Trial 1 composition (DM basis): Canola meal 37.6%, Soyplus^®^ 19.4%, Expeller meal^®^ 19.4%, Kinetic^®^ 6.6%, RP mix (Blood meal) 6%, sodium bicarbonate 4%, calcium carbonate 2.3%, white salt 1.2%, AB20 Bentonite^®^ 1%, PHD High VTM^®^ 0.9%, urea 0.6%, Mepron^®^ 0.5%, magnesium oxide 0.4%, Diamond V XPC^®^ 0.2%, Rumensin^®^ 0.1%. ^2^ Premix Trial 2 composition (DM basis): Ground corn 42%, soybean meal 14%, Soyplus^®^ 13%, corn gluten feed 9%, soybean hulls 4.7%, whole cotton seed 5.1%, calcium Ca 2.9%, molasses 2.3%, sodium bicarbonate 2.2%, bentonite 0.8%, Palmit 80^®^ 0.7%, magnesium oxide 0.6%, salt 0.6%, potassium carbonate 0.5%, potassium chloride 0.5%, urea 0.4%, trace mineral and vitamin mix 0.38%.

**Table 4:** Rumen FA profile of lactating cows fed the dietary treatments in Trial 1.

FA, g/100 g of Total FA	Treatment ^1^				SEM ^2^	*p*-Value ^3^		
	CO	UF	TF	TR		1	2	3
C14:0	1.10	1.11	1.07	1.15	0.04	NS ⁵	NS	NS
C15:0	0.61	0.60	0.61	0.67	0.02	NS	NS	NS
C16:0	27.8	26.8	26.8	25.4	0.8	NS	NS	NS
C17:0	0.40	0.38	0.39	0.39	0.01	NS	NS	NS
C18:0	31.7	20.9	23.3	24.5	2.5	0.03	NS	NS
*trans*-6/8 C18:1	0.54	0.96	0.95	1.05	0.09	0.01	NS	NS
*trans*-9 C18:1	0.28	0.75	0.70	0.77	0.12	0.02	NS	NS
*trans*-10 C18:1	1.16	3.96	3.84	3.64	0.95	0.07	NS	NS
*trans*-11 C18:1	2.57	4.44	4.04	4.30	0.4	0.02	NS	NS
*trans*-12 C18:1	0.79	1.11	1.19	1.25	0.06	<0.01	0.18	NS
Total trans C18:1	5.35	11.21	10.72	11.01	1.2	0.01	NS	NS
C18:1n-9	7.86	9.32	8.58	8.22	0.40	0.14	0.13	NS
C18:2n-6	9.43	10.92	9.74	9.33	1.2	NS	NS	NS
*cis*-9, *trans*-11 C18:2 CLA	0.068	0.132	0.128	0.123	0.02	0.05	NS	NS
*trans*-10, *cis*-12 C18:2 CLA	0.050	0.072	0.059	0.077	0.02	NS	NS	NS
*cis*-9, *cis*-12, *cis*-15 C18:3n-3	2.67	3.20	2.66	2.69	0.42	NS	NS	NS
C20:5n-3 EPA	N/D ⁴	0.54	0.2	0.14	0.2	0.18	0.14	NS
C22:5n-3	N/D	0.071	0.02	0.002	0.03	NS	NS	NS
C22:6n-3 DHA	N/D	0.44	0.23	0.26	0.10	0.05	0.19	NS
Total n-3	N/D	1.05	0.45	0.4	0.29	0.13	0.15	NS

^1^ CON = Control with no capsule (CO); UF = Control plus 200 untreated capsules per cow/day and mixed with the TMR; TR = Control plus 200 per cow/day of treated capsules placed directly into the rumen; TF = Control plus 200 treated capsules per cow/day and mixed with the TMR. ^2^ Standard error of the mean. ^3^ *p* ≤ 0.2 are shown for the contrasts: 1 = control vs. fish oil capsules (UF + TR + TF); 2 = untreated fish oil fed directly into the rumen (UF) vs. treated fish oil capsules (TR + TF); 3 = treated fish oil capsule fed directly into the rumen (TR) vs. treated fish oil capsule mixed with the TMR (TF). ⁴ N/D = non-detectable values. ⁵ NS = not significant (*p* ≥ 0.20).

**Table 6:** Plasma FA profile of lactating cows fed the dietary treatments in Trial 2.

FA, g/100 g of Total FA	Treatment ^1^			SEM ^2^	*p*-Value ^3^		Breed ^4^		SEM ^2^	*p*-Value ^3^	
	CON	UC	TC		1	2	H	J			
C14:0	0.31	0.33	0.32	0.02	NS ⁵	NS	0.33	0.3.0	0.01		NS
C16:0	6.2	6.5	6.1	0.24	NS	NS	6.5	6.0	0.25		NS
C18:0	10.2	9.6	9.3	0.34	0.12	NS	9.7	9.7	0.28		NS
*cis*-9 C18:1	3.5	3.1	3.1	0.17	0.04	NS	3.0	3.4	0.18		NS
*cis*-9, *cis*-12 C18:2n-6	37.7	39.2	37.0	1.58	NS	0.15	40.4	35.6	1.90		0.15
*cis*-9, *cis*-12, *cis*-15 C18:3n-3	2.1	2.0	2.0	0.11	NS	NS	2.1	1.9	0.13		NS
C20:4n-6	1.75	1.24	1.13	0.20	<0.01	NS	1.58	1.17	0.27		NS
C22:6n-3 DHA	0.12	0.23	0.18	0.04	0.18	NS	0.17	0.18	0.03		NS

^1^ CO = Control with no capsules; UC = Control plus 180 untreated capsules per cow/day; TC = Control plus 180 treated capsules per cow/day. ^2^ Standard error of the mean (highest when uneven samples). ^3^ *p* ≤ 0.2 are shown. The contrast: 1 = Control vs. fish oil capsules (UC + TC); 2 = untreated fish oil capsules (UC) vs. treated fish oil capsule (TC). ^4^ H = Holstein; J = Jersey. ⁵ NS = not significant (*p* ≥ 0.20).

**Table 7:** Milk FA profile of lactating cows fed the dietary treatments in Trial 1.

FA, g/100 g of Total FA	Treatment ^1^				SEM ^2^	*p*-Value ^3^		
	CO	UF	TF	TR		1	2	3
C4:0	4.4	4.33	4.21	4.23	0.14	NS ⁴	NS	NS
C6:0	2.22	2.09	2.01	1.95	0.08	0.05	NS	NS
C8:0	1.22	1.15	1.11	1.04	0.05	0.07	NS	NS
C10:0	2.75	2.61	2.52	2.31	0.12	0.07	NS	NS
C12:0	3.14	3.02	2.97	2.74	0.12	0.13	NS	NS
C14:0	10.4	10.2	10.2	9.8	0.21	NS	NS	NS
C15:0	0.89	0.87	0.82	0.86	0.03	0.14	NS	NS
C16:0	33.7	31.4	31.0	31.2	0.35	<0.01	NS	NS
C17:0	0.42	0.43	0.43	0.43	0.01	NS	NS	NS
C18:0	7.64	6.84	7.35	6.8	0.27	0.06	NS	NS
*trans*-5 C18:1	0.0073	0.018	0.021	0.0069	0.004	0.12	NS	0.06
*trans*-6/8 C18:1	0.37	0.61	0.57	0.62	0.026	<0.01	NS	NS
*trans*-9 C18:1	0.20	0.45	0.42	0.48	0.03	<0.01	NS	NS
*trans*-10 C18:1	1.29	2.64	2.54	2.39	0.53	0.07	NS	NS
*trans*-11 C18:1	0.93	1.84	1.54	1.58	0.11	<0.01	NS	NS
*trans*-12 C18:1	0.53	0.82	0.80	0.81	0.019	<0.01	NS	NS
*cis*-9 C18:1	15.7	14.5	15.8	16.2	0.40	NS	<0.01	NS
*cis*-9, *cis*-12 C18:2n-6	2.45	2.65	2.71	2.72	0.05	<0.01	NS	NS
*cis*-9, *trans*-11 C18:2 CLA	0.54	0.98	0.83	0.93	0.031	<0.01	0.02	0.08
*trans*-10, cis-12 C18:2 CLA	0.0063	0.0013	0.0013	0.012	0.003	NS	NS	0.06
*cis*-9, *cis*-12, *cis*-15 C18:3n-3	0.53	0.59	0.60	0.62	0.02	<0.01	NS	NS
C20:5n-3 EPA	0.03	0.073	0.066	0.053	0.01	<0.02	NS	NS
C22:5n-3	0.04	0.071	0.062	0.061	0.05	NS	NS	NS
C22:6n-3 DHA	0.017	0.095	0.083	0.102	0.01	0.05	NS	NS
Total n-3	0.088	0.238	0.211	0.216	0.04	<0.01	NS	NS

^1^ CON = Control with no capsule (CO); UF = Control plus 200 untreated capsules per cow/day and mixed with the TMR; TR = Control plus 200 per cow/day of treated capsules placed directly into the rumen; TF = Control plus 200 treated capsules per cow/day and mixed with the TMR. ^2^ Standard error of the mean. ^3^ *p* ≤ 0.2 are shown for the contrasts: 1 = control vs. fish oil capsules (UF + TR + TF); 2 = untreated fish oil fed directly into the rumen (UF) vs. treated fish oil capsules (TR + TF); 3 = treated fish oil capsule fed directly into the rumen (TR) vs. treated fish oil capsule mixed with the TMR (TF). ⁴ NS = not significant (*p* ≥ 0.20).

## Data Availability

Not applicable.
